# Hierarchical Spatio-Temporal Modeling of Naturalistic Functional Magnetic Resonance Imaging Signals via Two-Stage Deep Belief Network With Neural Architecture Search

**DOI:** 10.3389/fnins.2021.794955

**Published:** 2021-12-08

**Authors:** Yudan Ren, Shuhan Xu, Zeyang Tao, Limei Song, Xiaowei He

**Affiliations:** School of Information Science and Technology, Northwest University, Xi’an, China

**Keywords:** naturalistic fMRI, deep belief network, neural architecture search, hierarchical organization of brain function, functional brain network (FBN)

## Abstract

Naturalistic functional magnetic resonance imaging (NfMRI) has become an effective tool to study brain functional activities in real-life context, which reduces the anxiety or boredom due to difficult or repetitive tasks and avoids the problem of unreliable collection of brain activity caused by the subjects’ microsleeps during resting state. Recent studies have made efforts on characterizing the brain’s hierarchical organizations from fMRI data by various deep learning models. However, most of those models have ignored the properties of group-wise consistency and inter-subject difference in brain function under naturalistic paradigm. Another critical issue is how to determine the optimal neural architecture of deep learning models, as manual design of neural architecture is time-consuming and less reliable. To tackle these problems, we proposed a two-stage deep belief network (DBN) with neural architecture search (NAS) combined framework (two-stage NAS-DBN) to model both the group-consistent and individual-specific naturalistic functional brain networks (FBNs), which reflected the hierarchical organization of brain function and the nature of brain functional activities under naturalistic paradigm. Moreover, the test-retest reliability and spatial overlap rate of the FBNs identified by our model reveal better performance than that of widely used traditional methods. In general, our model provides a promising method for characterizing hierarchical spatiotemporal features under the natural paradigm.

## Introduction

Neuroscientists have long realized that functional brain networks (FBNs) present varying degrees of activation responses in a multi-scale hierarchical structure (Ferrarini et al., 2009). In the past, most of these studies used task-based paradigms to explore the hierarchy of brain network, which are designed to engage and isolate a particular aspect of brain function such as motor or visual perception. However, it is unclear whether and to what extent such task paradigms could uncover the complex mental processes in real life. On the other hand, a majority of studies on FBN/functional connectivity have relied on resting-state paradigm, which requires low level of performance demand and thus becomes a popular tool to investigate the FBNs and clinical populations. However, due to its unconstrained nature, resting state suffers from unwanted behavioral confounds such as head motion and microsleep (Vanderwal et al., 2015). To address limitations of traditional task and resting state paradigms, recent studies employ naturalistic paradigms, such as movie viewing, which examine the complex neural processes during dynamic, naturally engaging stimuli that greatly resembles the brain function under real-life condition (Sonkusare et al., 2019). However, brain activities under naturalistic paradigms are always dynamic and complex with their distinctive properties (Sonkusare et al., 2019), thus causing difficulty to model their neural correlates and awaiting appropriate computational framework.

In previous studies, a variety of conventional methods for modeling fMRI data to reconstruct and characterize FBN have been developed, such as general linear model (GLM) (Beckmann et al., 2003), independent component analysis (ICA) (Calhoun and Adali, 2006), and sparse dictionary learning (SDL) (Lv et al., 2015). Although these methods can construct meaningful FBNs, due to their superficial nature, they may not be able to detect hierarchical FBNs and temporal features, which is an important nature for FBNs (Meunier et al., 2009; Qiang et al., 2020b). In recent years, deep learning models have been widely used to model fMRI data, due to their powerful representation and abstraction capabilities, including convolutional neural network (CNN) (Zhao et al., 2020), deep variational auto-encoder (DVAE) (Qiang et al., 2020a), deep sparse recurrent auto-encoder (DSRAE) (Li et al., 2021a), deep convolutional autoencoder (DCAE) (Huang et al., 2018; Zhao et al., 2021), and deep belief network (DBN) (Zhang et al., 2019b; Qiang et al., 2020b). These studies revealed that these deep learning models significantly outperformed the traditional shallow models in extracting meaningful hierarchical FBNs and temporal features from fMRI data. For instances, Huang et al. used DCAE model to learn medium and high-level features from task-based fMRI (tfMRI) time series (Huang et al., 2018). Qiang et al. (2020b) employed DBN to model the volumetric tfMRI data achieving great performance in characterizing the task-based functional activations.

However, although these models have exhibited performance in extracting hierarchical spatiotemporal features of fMRI data at multiple scales, there still remain big challenges in such deep leaning models. Firstly, due to the high dimensionality of fMRI data and a variety of training parameters, manual design of neural architecture depending on experience is very time-consuming and less reliable. Hence, it is necessary to develop computational framework for automatic construction of optimal neural architecture (NA) for FBNs construction models. Recently, there have been some literature studies that successfully applied neural architecture search (NAS) framework to fMRI data for brain network modeling (Zhang et al., 2019a; Qiang et al., 2020b; Li et al., 2021b,c). For instance, Zhang et al. (2019a) has proposed a NASNet with DBN model to identify hierarchical spatio-temporal features from static task-based temporal fMRI data, and Qiang et al. (2020b) has proposed a NAS-vs. DBN framework to model the static task-based volumetric fMRI data. Specifically, a DBN model is typically stacked by multiple Boltzmann machine (RBM) (Fischer and Igel, 2012), which naturally act as an effective hierarchical feature extractor as a whole to extract hierarchical brain spatio-temporal features from each hidden layer. Although these studies revealed the superiority of DBN in characterizing hierarchical FBNs during task paradigm and the capability of NAS in automatic identification of neural architecture (NA), these models focused on uncovering the hierarchical task-based FBNs, still ignoring the significant nature of brain activities under naturalistic paradigm.

Secondly, various research studies have demonstrated the intrinsic properties of neural process under naturalistic paradigm, that brain responses evoked by this condition exhibit highly consistency across individuals, but also show great inter-subject variability, especially in heteromodal association cortices (Golland et al., 2007; Ren Y. et al., 2017), which reflects high degree of individuality and uniqueness in internal neural process. However, existing deep learning models on FBNs identification overlooked this important nature of naturalistic paradigm (Zhang et al., 2019a; Qiang et al., 2020b; Li et al., 2021b), thus awaiting suitable computational models that can uncover hierarchical temporal features and FBNs possessing group consistency and individual uniqueness simultaneously. Inspired by the previous successful applications of NAS-DBN framework and in order to solve the existing problems mentioned above, we proposed a novel two-stage DBN model with NAS (two-stage NAS-DBN framework) to extract the group-consistent and individual-unique temporal and spatial characteristics of NfMRI signals at multiscale, further suggesting the hierarchical organization of brain function under naturalistic paradigm. Furthermore, the effectiveness and reproducibility of our model were further evaluated against conventional methods, where our model exhibited improved reliability and overlap rate, thus offering a superior data-driven strategy for identifying brain function during naturalistic condition.

## Materials and Methods

**FIGURE 1:**
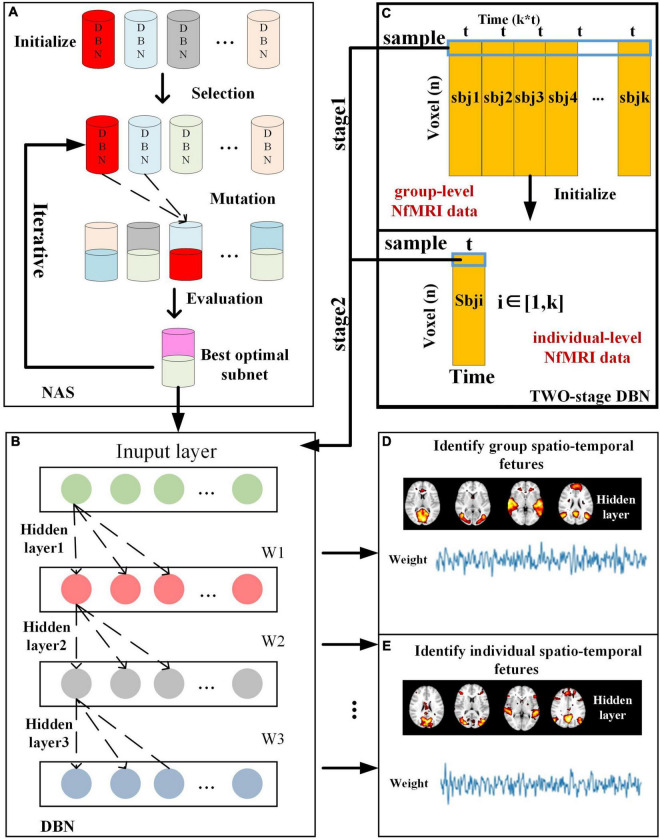
Illustration of computational framework of two-stage NAS-DBN model, including **(A)** NAS process, **(B)** DBN model training process, **(C)** group-level and individual-level DBN model, and **(D)** Identification of spatio-temporal features.

The computational framework of two-stage NAS-DBN is illustrated in Figure 1. The entire process of NAS was implemented based on group-level NfMRI data. First, 30 sub-nets were randomly initialized. Some potential sub-nets were selected as particles to represent specific network architecture of DBN model (Figure 1A). Reconstruction error of each sub-net was calculated after training. Then, evaluation, mutation and updating operation were conducted iteratively (Figures 1A,B). After NAS, DBN with the optimal neural architecture was first trained on group-level NfMRI data (Figure 1C), resulting in group-level spatial FBNs and temporal features (Figure 1D). Afterward, we further trained the individual-level DBN model initialized by group-level DBN results for each subject’s NfMRI data (Figure 1C), producing individual-level spatial FBNs and temporal features with group correspondence (Figure 1E).

### Experimental Data and Preprocessing

Seventeen right-handed healthy subjects participated in the study. During scan, participants freely watched a 20-min movie named “The Butterfly Circus,” which depicts a story of a man born without limbs who is encouraged by a famous circus performer to discover his potential. All the subjects signed a written informed consent and reported that they had not previously seen the movie. The experiments were composed of two scanning sessions with a 3-month interval, where all the subjects watched the same movie in each session (Sonkusare et al., 2019). We marked the datasets obtained from the two sessions as session A and session B, respectively.

fMRI images were acquired from a whole-body 3T Siemens Trio MRI scanner with following scanning parameters: TR = 2,200 ms, TE = 30 ms, FA = 79°, FOV = 134 mm × 134 mm, a 64 × 64 acquisition matrix, 44 axial slices, and 3 mm^3^ isotropic voxels. The preprocessing pipeline was conducted by Statistical Parametric Mapping toolbox (SPM12), including slice timing correction and motion correction, co-registration, normalization to 2 mm MNI152 standard template (The Montreal Neurological Institute), spatial smoothing with 6 mm full width half maximum Gaussian kernel, band pass filtering (0.0085 ± 0.15 Hz), and masking. The fMRI signals of each subject contain 530 volumes. Whole-brain fMRI signals of each subject were extracted and stacked into a 2D matrix *S*_*x*_ (The *S*_*x*_ represents the 2D fMRI signal matrix of each subject, where *x* represents the label of subject.), where each column represents the fMRI signals of each voxel. In addition, fMRI time series of each voxel were normalized to have zero mean and unit variance. We concatenated all individual subjects’ fMRI time series to a group-wise matrix for group-level DBN training.

### Neural Architecture Search Framework

As the particle swarm optimization algorithm (PSO) owns multiple advantages (Kennedy and Eberhart, 1995), including less parameter requirements, simple formula, and easy to implement, we applied a PSO-based NAS framework to search for the optimal neural architecture (NA) for our two-stage DBN model. Specifically, we first randomly generated 30 sub-nets with different NAs, which consisted of most critical hyperparameters: the number of nodes and hidden layers. In order to increase the diversity of sub-nets, some potential sub-nets will be selected after initialization and performed mutation, to generate new generation of particles based on the idea of aging evolution (Real et al., 2019). The sub-net after mutations will be mapped to the position of each particle. According to the principle of PSO algorithm, all the particles were evaluated by a fitness function, which was defined by the reconstruction error of DBN (Kennedy and Eberhart, 1995). This NAS procedure was conducted iteratively. Each iteration recorded the current global optimal NA with the most accurate reconstruction in that iteration and replaced the original one (Kennedy and Eberhart, 1995). Finally, after all the iterations, a single particle owning the most accurate reconstruction was selected as global optimal NA. Specifically, the mutation strategy of PSO algorithm is defined as follows:


(1) $$v_{i}^{h+1}=w*v_{i}^{h}+c_{1}*{\mathrm{Rand}}_{1}\,\left({\mathrm{pbest}}_{i}^{h}-{\mathrm{nas}}_{i}^{h}\right)+c_{2}*{\mathrm{Rand}}_{2}\,\left({\mathrm{gbest}}_{i}^{h}-{\mathrm{nas}}_{i}^{h}\right)$$

(2) $${\mathrm{nas}}_{i}^{h+1}={\mathrm{nas}}_{i}^{h}+v_{i}^{h+1}$$

In Equations (1) and (2), $n\,a\,s_{i}^{h}$ and $n\,a\,s_{i}^{h+1}$ indicate current and next search of neural architecture, and $v_{i}^{h}$ and $v_{i}^{h+1}$ are the current and next mutative velocity to update the NA. The subscript *h* and *I* represents current iteration/generation; *w* is the inertia weight; *c*_*1*_and *c*_*2*_ are constant real values; *Rand*_*1*_ and *Rand*_*2*_ are random real numbers; $p\,b\,e\,s\,t_{i}^{h}$ represents historical optima for each sub-net during iterations, and $g\,b\,e\,s\,t_{i}^{h}$ is global optimal NA. The above constant values are set as the default value according to previous study (Kennedy and Eberhart, 1995). Due to GPU memory limitations, the search range of nodes is set to [100, 800], the search range of layers is set at [2, 10].

### Two-Stage Deep Belief Network-Based Model

In general, DBN is composed of multiple stacked Restricted Boltzmann Machines (Hinton et al., 2006), which models the latent distribution of input data through interactions between visible and hidden variables, and of which structure makes it suitable to extract the hierarchical features of fMRI data. DBN consists of visible layer variable *v* and hidden layer variable *h*, of which energy function is as follows:


(3) $$E\,\left(v,h\right)=\sum b_{i}\,v_{i}-\sum b_{j}\,h_{j}-\sum v_{j}\,h_{j}\,w_{\mathrm{ij}}$$

Where *v*_*i*_ and *h*_*j*_ represents the activation state of two layers; *b*_*i*_ and *b*_*j*_ indicate their bias; *w*_*ij*_ is the weight between two layers. In this work, the DBN model with the optimal NA was applied to define the architecture of DBN model, aiming to characterize the two-stage hierarchical FBNs and corresponding temporal features. In the first stage, the optimized DBN was adopted to model group-level NfMRI data to train a weight matrix from each layer as *W*_*i*_ ∈ *R*^*kt*×*m*^, which represents the group-wise temporal features. Specifically, all the individual fMRI signals were concatenated along the time to a group-level NfMRI matrix for first-stage training. In the second stage, the subject-specific DBN models were applied to each individual NfMRI matrix to further identify individual-level temporal features and FBNs while maintaining their correspondence across subjects. To achieve this, the group-level weight matrix *W*_*i*_ of each layer derived from first stage was used to initialize the subject-specific DBN model. In general, according to previous FBNs identification literatures (Hu et al., 2018; Zhang et al., 2019a), extracting FBNs from fMRI signal matrix using DBN/RBM model can be regarded as a blind source separation problem, which shares similar structures with matrix factorization problem in terms of the relationship among the observed fMRI data, latent temporal features, and spatial maps. Thus, in our model, NfMRI temporal signals matrix can be decomposed as the temporal features and spatial maps via DBN model. While the weight matrix of each layer in DBN was regarded as temporal features, the output of hidden layer represented the corresponding spatial maps of these temporal features that can be mapped back to original 3D brain image space, for both group-level and individual-level DBN models.

### Compare With Widely Used Traditional Methods

While previous studies have shown that widely used data-driven methods such as ICA and SDL are effective to identify typical FBNs (such as Resting state network, RSN), our model overcomes the weakness of these shallow models in terms of extracting the complex interactions between brain regions and the hierarchical organization of FBNs. Thus, to evaluate the performance of the proposed two-stage DBN model, we compared it to ICA and SDL in terms of modeling fMRI time series to construct FBNs. Both group ICA and SDL were applied to spatial-concatenated group-wise NfMRI signals of all the subjects. Specifically, SDL was implemented by effective online dictionary learning method via a public software^1^, which decomposes the group-wise fMRI signals into dictionary matrix D and coefficient matrix A (Mairal et al., 2010). Note that D is commonly shared by all subjects and represents extracted temporal features, and the A has the same spatial voxel organization and group correspondence of input signals and thus is composed of individual-level FBNs of each subject. Each coefficient vector in A can be back into the 3D brain space to derive FBN corresponding to each dictionary. In addition, ICA was implemented by fast ICA toolbox (Hyvarinen, 1999), which decomposes the 2D group-wise NfMRI signal matrix into a set of representative temporal features and corresponding individual-level spatial patterns. A statistical coefficient mapping method was adopted to individual-level FBNs to derive group-level statistical coefficient maps (z-score maps) for comparison across different methods, according to our previous study (Ren Y.D. et al., 2017). For fare comparison, the number of components for SDL and ICA were set as same as the node number of our modal, and all the FBNs derived by three methods were using a threshold of *z* > 1.65 after being transformed to the standard z-score.

To evaluate the performance of three models in terms of effectiveness of constructing spatial patterns, quantitatively, we compared the spatial similarity between spatial patterns (FBNs) derived by these models and the well-established RSNs, where the spatial pattern overlap rate O is defined as


(4) $$O\,\left(S,T\right)=\frac{\left|S\,\cap T\right|}{\left|T\right|}$$

where S is the FBN identified by these methods and T is the RSN template, respectively.

### Inter-Subject Correlation Analysis for Temporal Features

To investigate the hierarchical organization of temporal responses derived from proposed framework, we calculated the inter-subject correlation (ISC) using individuals’ temporal responses extracted from weight matrix of DBN model, where ISC measures the inter-subject consistency for temporal responses of each atom from each hidden layer across individuals (Hasson et al., 2004; Nastase et al., 2019). Specifically, individual-level ISC value of an atom for subject *i* is defined as following equation.


(5) $${\mathrm{ISC}}_{i}=\frac{1}{N-1}\,\sum_{j=}^{N}r_{\mathrm{ij}},\mathrm{where}\,i,j=1,2,\ldots ,17;i\neq j.$$

Where *r*_ij_ represents the Pearson correlations between the temporal response corresponding to one atom in one subject *i* and that temporal responses in each of the remaining subjects *j* in the group. This ISC analysis was conducted for each atom of each hidden layer for all the subjects, separately. We then derived the ISC metric for each layer and each subject by averaging the ISC values across all the atoms belonging to the same layer, respectively. Moreover, the group-level ISC metric was derived by averaging all the individual’s ISCs for each layer, respectively.

### Test-Retest Reliability Analysis for Functional Brain Networks

To test the reproducibility of each brain network measures derived from our model and commonly-used methods, we conducted the same two-stage DBN, group-wise SDL and ICA on fMRI dataset of session B, and detected matching FBNs, which share maximum number of overlapping voxels with FBNs derived from session A. In addition, further careful manual inspection was conducted to verify the matching FBNs of the two sessions. Then, we calculated test-retest reliability of each selected matching FBNs. The reliability is quantified by calculating the intra-group correlation coefficient (ICC) between two datasets (Shrout and Fleiss, 1979; Mcgraw, 1996), which is defined as following equation.


(6) $$\mathrm{ICC}=\frac{{\mathrm{MS}}_{P}-{\mathrm{MS}}_{e}}{{\mathrm{MS}}_{P}+\left(d-1\right)\,{\mathrm{MS}}_{e}}$$

The details of this procedure were accomplished according to our previous study (Ren Y.D. et al., 2017). We evaluated the test-retest reliability of FBNs at voxel-wise level according to previous study (Guo et al., 2012). The test-retest reliability is divided into five levels, including excellent (ICC > 0.8), good (ICC 0.6–0.79), moderate (ICC 0.4–0.59), fair (ICC 0.2–0.39), and poor (ICC < 0.2).

## Results

### Two-Stage Neural Architecture Search-Deep Belief Network Implementation

To quantitively evaluate the effectiveness and reproducibility of NAS framework and obtain the reliable optimal architecture of DBN model, we independently performed 10 times of NAS processes. Considering subjects and stimuli of the two sessions are the same, we only used 17 individuals of session A as training dataset for NAS procedure.

**FIGURE 2:**
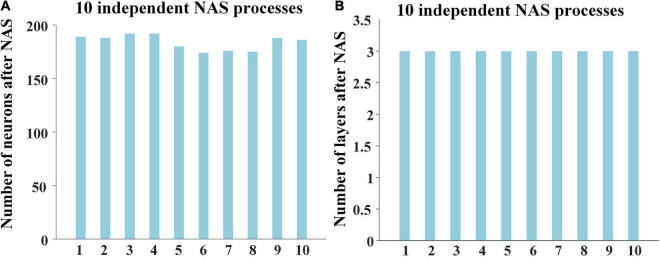
Results of 10 independent NAS processes, including **(A)** the numbers of neurons and **(B)** the numbers of layers of 10 independent NAS processes.

For the 10 results of NAS, the numbers of neurons were in range from 170 to 200, and layers were always 3, showing high consistency and robustness (Figure 2). The reconstruction errors of third layer after performing NAS were less than 10^–7^. These experimental results show the robust superiority of our NAS framework. Afterward, we averaged 10 NAS results, thus determining that the optimal architecture of DBN model has 3 layers and 184 neurons, which was further used for characterizing both group-level and individual-level hierarchical FBNs on session A and session B separately.

For the hyperparameters of two-stage DBN model, as each individual NfMRI signal has 530 volumes, the number of visible units of group-level DBN model in the first stage is 9,010 (530 × 17) for both session A and session B. In addition, the number of neurons and layers in the first-stage DBN were determined according to results of NAS. The target sparsity (the ratio of active hidden units) and learning rate in the first/second/third hidden layer were set as 0.01/0.05/0.05 and 0.001/0.001/0.001, respectively, and the batch size was set as 10. The model converged after approximately 50 epochs. In the second-stage, the individual DBN model was applied to 17 subjects with 530 visible units, separately. The hidden units and other hyperparameters set in the second stage of DBN were set as same as the first stage. In addition, all the experimental results were run on both session A and session B, including group-level and individual-level FBNs identification, ISC analysis of temporal features, and spatial/temporal hierarchy analysis. As two sessions yielded similar results, we thus mainly presented results based on session A in the main text, and results based on session B in the supplementary results as a further validation (Supplementary Figures 1–5). The code was designed based on the Deepnet framework^2^ and ran on a deep learning server with GeForce GTX 1080 TI. The NAS process was accomplished on one GPU card within acceptable time (8 h).

### Identified Group-Level and Individual-Level Hierarchical Functional Brain Networks

**FIGURE 3:**
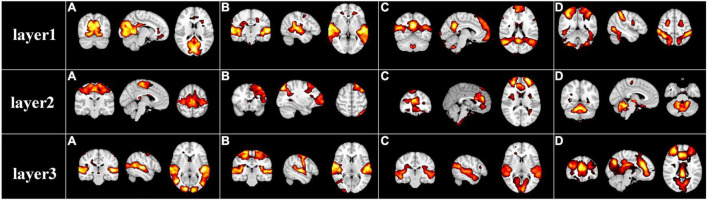
Representative group-level functional brain networks from three layers identified by first-stage DBN model based on session A. Layer 1: **(A)** visual network, **(B)** auditory network, **(C)** default mode network, and **(D)** dorsal attention network. Layer 2: **(A)** sensorimotor network, **(B)** frontoparietal network, **(C)** executive control network, and **(D)** cerebellum network. Layer 3: **(A)** visual-auditory network, **(B)** auditory-sensorimotor network, **(C)** visual-auditory network, and **(D)** visual-executive control network.

After the specification of the model architecture, we further explored the hierarchical FBNs during movie viewing defined by the our two-stage NAS-DBN framework. We first selected and showed some representative hierarchical group-level FBNs identified in the first stage DBN. Some well-known brain networks can be successfully identified (Figure 3), including the visual network [layer1(a)], auditory network [layer1(b)], default mode network [layer1(c)], dorsal attention network [layer1(d)], sensorimotor network [layer2(a)], frontoparietal network [layer2(b)], executive control network [layer2(c)] cerebellum network [layer2(d)]. In addition to those well-established networks, our framework can also identify some complex/interactive FBNs, which appear to reveal the functional interactions between different brain regions/networks, such as auditory-sensorimotor network [layer3(b)], visual-auditory network [layer3(a), (c)], visual-executive control network [layer3(d)]. Interestingly, while simple or well-established FBNs are derived from shallower layers of DBN model, the complex interactive FBNs composed of different brain networks are found in deeper layers, which suggests the hierarchical organization of FBNs under naturalistic condition. Similar group-level results were found on session B which were illustrated in Supplementary Figure 1.

**FIGURE 4:**
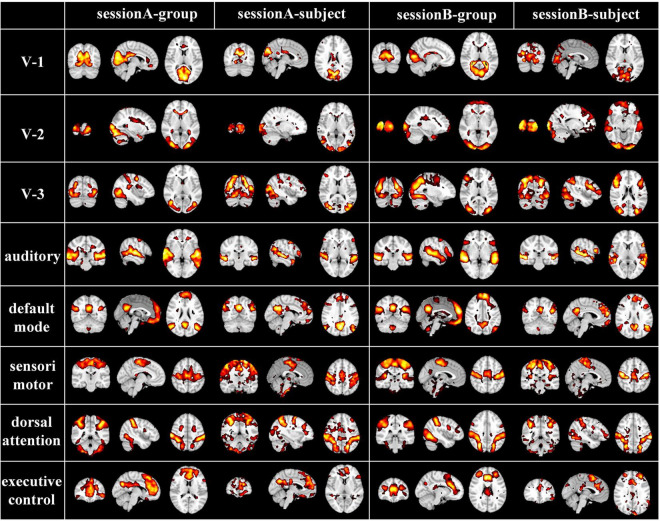
Corresponding group-level and individual-level FBNs in an exemplar subject for two sessions (V-1: medial-visual network, V-2: occipital pole-visual network and V-3: lateral-visual network).

While naturalistic stimuli can trigger highly consistent neural responses in primary sensory areas, neural activities evoked by this condition especially in the higher-order heteromodal cortices also show great inter-subject variability, which can be modeled by our individual-level DBN model with cross-subject correspondences kept by group-level DBN model. To further verify the correspondence of group-level and individual-level FBNs, we randomly selected and compared representative group and individual FBNs in an exemplar subject, including three visual networks, auditory network, default mode network, sensorimotor network, dorsal attention network, executive control network (Figure 4). Specifically, the FBNs derived from two stages correspond well, where most of the spatial patterns learned in the first stage are perfectly preserved in individuals FBNs with individual specific variability maintained. This further demonstrated that our model can effectively characterize meaningful spatial patterns with well-established correspondence between group-level and individual-level FBNs, keeping the important properties of FBNs under naturalistic condition.

### Hierarchical Organization of Spatio-Temporal Patterns Under Naturalistic Condition

DBN model consists of multiple stacked RBM block, which makes it a feature extractor to capture the hierarchical temporal and spatial features from NfMRI data. In general, the hierarchical temporal features can be derived from weight matrix and the associated spatial features can be represented by the output of each hidden layer of DBN model, respectively. Specifically, our two-stage NAS-DBN framework can not only characterize hierarchical temporal responses, but also reveal the meaningful group-level and individual-level FBNs with hierarchical organization.

#### Hierarchical Temporal Organization Revealed by Inter-Subject Correlation

**FIGURE 5:**
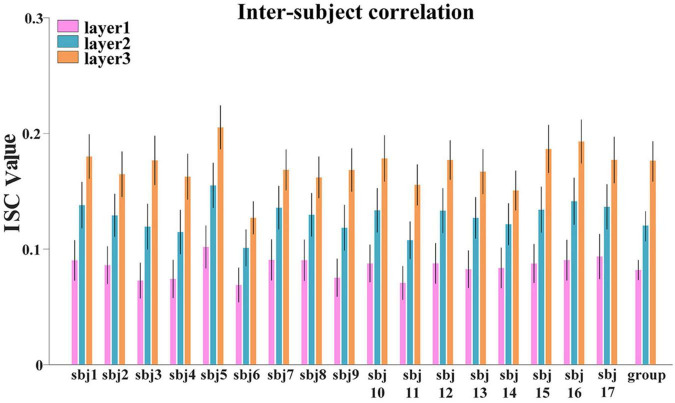
The ISC metric in each layer at individual-level and group-level for session A. Error bar indicates standard error of mean (SEM).

First, to investigate the hierarchical organization of temporal responses derived from proposed framework, we measured and compared the inter-subject correlation (ISC) of individuals’ temporal responses, at both individual-level and group-level. We averaged the ISC metrics for each layer at individual-level and group-level, respectively, the lower layer temporal responses show less inter-subject consistency, while higher layer temporal responses show higher consistency across individuals at both individual-level and group-level (Figure 5), which reveals the existence of hierarchical organization of naturalistic temporal features. Consistent results were found in session B and shown in Supplementary Figure 2

**FIGURE 6:**
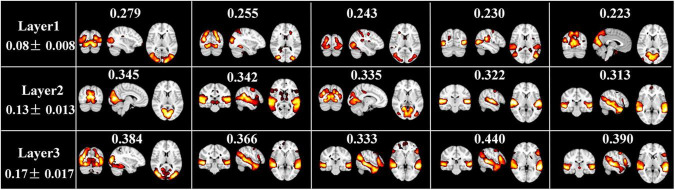
Top 5 spatial maps with highest ISC metrics at each layer. ISC value corresponding to each FBN, and the average and standard deviation ISC value of each layer are labeled.

In addition, Figure 6 illustrates the top five FBNs with highest group-level ISC in each layer. Most of these FBNs are localized in primary sensory cortices, especially in visual or auditory network, which are consistent with ISC map derived from naturalistic fMRI studies (Nastase et al., 2019), where most brain regions show similar neural responses among subjects during movie viewing. Furthermore, this interesting experimental result reflected excellent performance of our model in mining temporal features under natural viewing condition.

**FIGURE 7:**
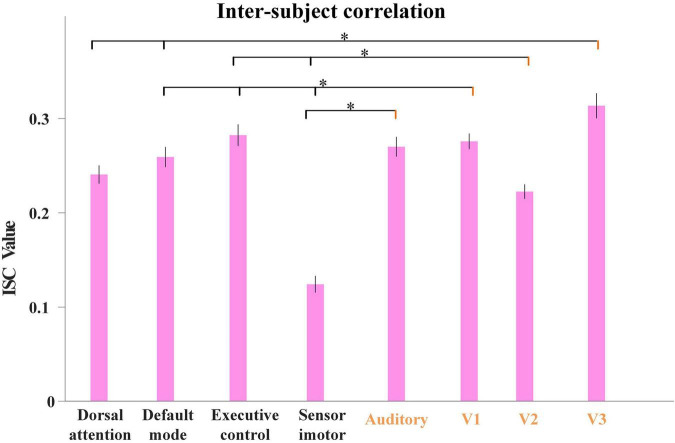
The average ISC values from individual temporal features corresponding to each FBN for session A, including dorsal attention network, default-mode network, executive control network, sensorimotor network, auditory network, medial-visual network (V1), occipital pole-visual network (V2) and lateral-visual network (V3). The error bar refers to standard error of mean (s.e.m). The statistical test was conducted by ANOVA, where * represents FDR-corrected *p* < 0.001 (FDR: False discovery rate).

Inspired by this, as our proposed model characterized group-level and individual-level FBNs with good correspondence, we further calculated the average ISC values derived from all the individual temporal features corresponding to each FBN illustrated in Figure 4. As shown in Figure 7, we used Analysis of Variance (ANOVA) test to evaluate the differences in ISC value between FBNs, with session A as an example. Specifically, we can clearly find that the ISC values of visual network and auditory network are generally significantly higher than other FBNs, further indicating that neural responses in primary sensory areas are more consistent than heteromodal cortices triggered by naturalistic stimuli and the effectiveness of proposed model in detecting FBNs with this import properties. The results of session B are quite consistent and illustrated in Supplementary Figure 3.

#### Functional Brain Networks and Temporal Hierarchy: Spatial and Temporal Similarity Revealed by Inheritance Similarity Rate

We here further excavated the hierarchical organization of group-level FBNs and associated temporal features between adjacent layers. To quantitatively measure the connection between hierarchical patterns of different layers, the inheritance similarity rate (ISR) between a lower layer spatial maps *N*^(*L*)^and a higher layer space maps *N*^(*H*)^ is defined as follows:


(7) $$\mathrm{ISR}\,\left(N^{\left(L\right)},N^{\left(H\right)}\right)=\frac{\sum_{i=1}^{n}\left|N_{i}^{\left(L\right)}\,\cap N_{i}^{\left(H\right)}\right|}{\sum_{i=1}^{n}\left(N_{i}^{\left(H\right)}\right)}$$

**FIGURE 8:**
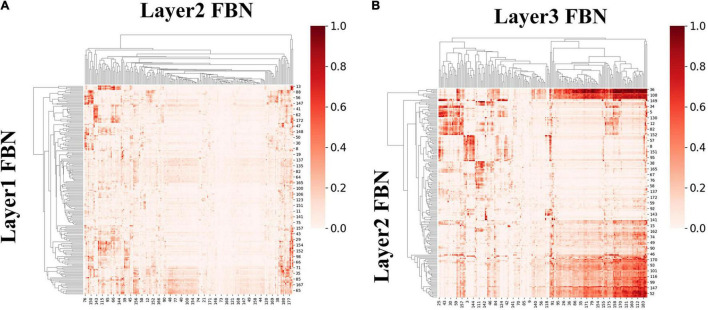
The group-level ISR maps of spatial maps across layers for session A. **(A)** Is the ISR between layer 1 and layer 2. **(B)** Is the ISR between layer 2 and layer 3.

We first calculated ISR metric for FBNs derived from first-stage DBN model for session A and session B, respectively. As two sessions yielded similar results, results for session B were listed in Supplementary Figure 4. Specifically, the group-level ISR for FBNs between layer 2 and layer 1 and that between layer 3 and layer 2 are shown in Figure 8. Note that the ISR between spatial patterns in higher layers shows more similarity than that between lower layers (two-sample *t*-test, *p* < 10^−307^), which indicates that FBNs in higher layer show more complexity and richness, further suggesting the existence of hierarchical structure of FBNs under naturalistic condition.

**FIGURE 9:**
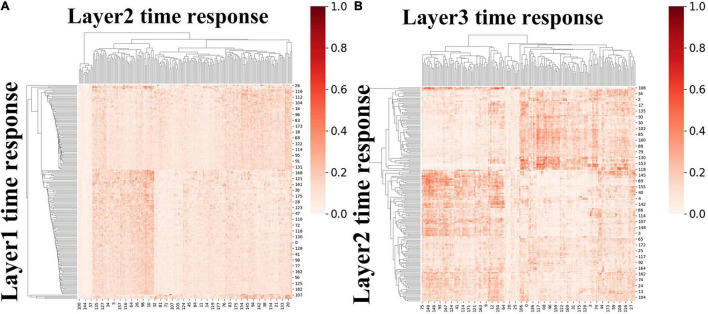
The group-level ISR maps of temporal responses across hidden layers for session A. **(A)** The ISR map between layer 2 and layer 1, and **(B)** the ISR map between layer 3 and layer 2.

Moreover, we further calculated ISR metrics for temporal responses derived from first-stage DBN model between adjacent layers for session A and session B, respectively. As two sessions yielded similar results, the results of session B were listed in Supplementary Figure 5. As shown in Figure 9, these results further validate the existence of temporal hierarchy extracted by the proposed model (two-sample *t*-test, *p* < 10^−11^). In general, these ISR maps between associated networks and temporal features between different layers quantitatively confirmed the hierarchical organization of spatial distributions and temporal features, further suggesting the superiority of our proposed model in modeling meaningful spatiotemporal features under naturalistic condition.

#### Comparison of Neural Architecture Search Two-Stage Deep Belief Network With Independent Component Analysis and Sparse Dictionary Learning

**FIGURE 10:**
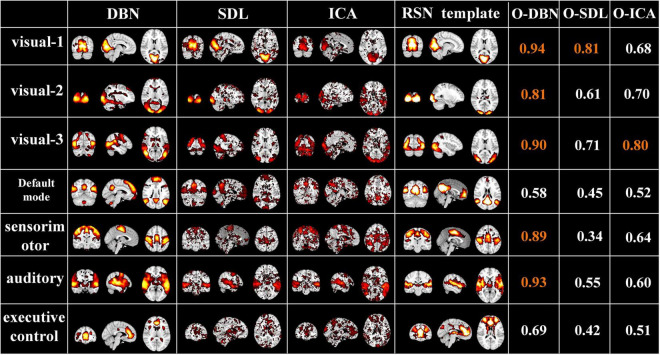
Representative FBNs defined by NAS-two stage DBN, SDL, and ICA, and the corresponding RSNs template. The right three columns represent the overlap rate of FBNs derived from each method. The overlap rate greater than 0.8 were marked in orange.

Finally, to evaluate the performance of the proposed two-stage DBN model, we compared it to widely used data-driven methods, ICA and SDL, in terms of overlap rate with RSN template and test-retest reliability of FBNs. As shown in Figure 10, we found that the overlap of the FBNs derived by our model are generally higher than that of ICA and SDL, calculated according to Equation (4). In particular, the overlap rate of auditory and visual networks, of which neural activities are strongly triggered by naturalistic stimuli, are higher than 0.8. In addition, while only few FBNs related to primary sensory cortices derived from ICA and SDL have overlap rate higher than 0.8, most of FBNs capture overlap rate lower than 0.6. This result demonstrated that our proposed framework can define meaningful spatial patterns during natural viewing condition than conventional data-driven methods.

**FIGURE 11:**
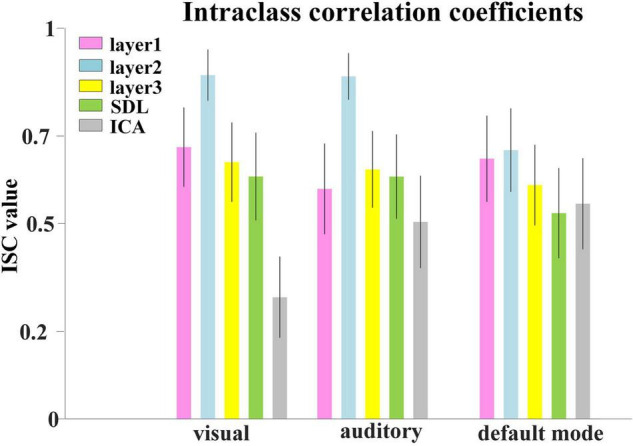
Test-retest reliability of FBNs. Average voxel-wise ICCs of networks detected by all the methods. Error bars signify standard error of mean (SEM).

**FIGURE 12:**
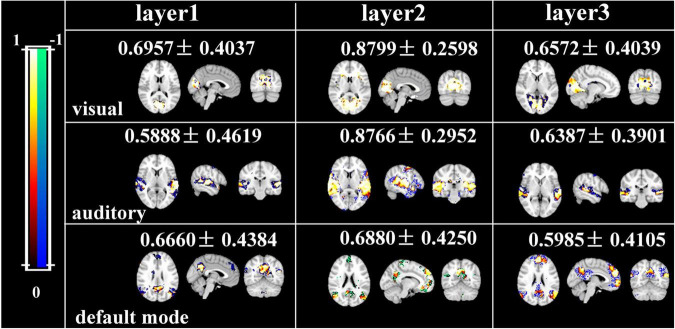
Brain maps of the voxel-wise ICCs of manually matching brain network using two-stage DBN.

Afterward, we would like to evaluate the long-term test-retest reliability of our method. Two-stage NAS-DBN, group ICA and SDL were employed to identify FBNs in the repeated scan sessions. To compare the test-retest reliability of these methods, we focused on three networks that can be defined in both sessions by all three methods, including visual network, auditory network and default mode network. We assessed the voxel-wise ICC of each FBN, where all three methods showed a range of reliability at the voxel level across different networks (Figure 11). Specifically, the voxel-level ICCs are either good or excellent in the visual network (layer1, 0.70; layer2, 0.88; layer3, 0.66) and default mode network (layer1, 0.67; layer2, 0.69; layer3, 0.60) defined by our method, but generally reduce to fair or moderate level with ICA (visual, 0.31; default mode, 0.55) and SDL (visual, 0.62; default mode, 0.53) methods. For the auditory network, average voxel-ICCs are at the moderate level in layer1 of DBN model (0.59), at the excellent or good level with layer2 (0.88) and layer3 (0.64), while ICCs are good in auditory network using SDL (0.62), and in moderate using ICA (0.50) method. In addition, we also visualized brain maps of the voxel-level ICCs of our model in Figure 12 and of ICA, SDL methods in Supplementary Figure 6. Moreover, to quantitatively assess the statistical differences of voxel-level ICCs derived from three methods, we applied two-sample *t*-tests with multiple testing corrections to ICCs of each representative network, respectively. Consequently, except that the ICCs of auditory network derived from layer1 of the proposed model are significantly lower than that derived from SDL (FDR-corrected *p* < 0.001), the ICCs of all the other networks derived from the proposed model are significantly higher than that derived from ICA and SDL methods (FDR-corrected *p* < 0.001), indicating the superiority of proposed model in characterizing spatial patterns with good long-term reliability under naturalistic paradigm (Figure 11).

## Conclusion

In this study, we proposed a two-stage NAS-DBN framework to derive group-level and individual-level spatio-temporal patterns from NfMRI signals, offering one of the first applications of NAS-DBN framework for analyzing dynamic naturalistic fMRI data. The advantages of our framework are summarized as follows. First, based on PSO, our NAS framework can find feasible optimal solution for neural architecture of DBN within acceptable time with limited computing resource. In addition, according to previous NAS-vs. DBN study (Qiang et al., 2020b), employing testing dataset in NAS process can potentially avoid overfitting problem, which can be realized using larger NfMRI dataset in the future work. Second, compared with other FBNs detection frameworks based on deep learning models, the proposed model can characterize hierarchical organization of FBNs and associated temporal features under naturalistic condition, which is an intrinsic nature of brain function and can be revealed by our experimental results. Third, compared with the simple NAS-DBN model, our two-stage NAS-DBN framework has been developed based on the critical properties of natural viewing condition, that is, neural processes under this condition exhibit highly consistency across individuals, and also show great inter-subject variability especially in heteromodal association regions (Golland et al., 2007; Ren Y. et al., 2017). Consequently, with well-established correspondence between two stages of DBN models, our framework offers potential advantage in characterizing group-level FBNs that reveal the consistency of neural processes across subjects and individual-level FBNs with cross-subject correspondence that maintain the subject specific variation and reflect high degree of individuality in internal neural process, verifying this critical property. Thus, compared with simple NAS-DBN model, our model provides a powerful tool for conducting inter-group/inter-subjects comparison of representative temporal features/FBNs. Finally, based on comprehensive comparisons with ICA and SDL methods in terms of network identification and test-retest reliability analyses, the experimental results demonstrated that our model could be more effective and reliable in identifying FBNs during naturalistic paradigms, where good long-term test-retest reliability is a necessary feature of a successful FBN biomarker for clinical study (Guo et al., 2012). With the superiority of inter-group/subject correspondence and meaningful FBNs with good long-term reliability established by two-stage NAS-DBN model, our framework could potentially be useful in clinical research, to elucidate abnormal brain function and develop neuroimaging markers for neuropsychiatric disorders.

## Data Availability Statement

The original contributions presented in the study are included in the article/Supplementary Material, further inquiries can be directed to the corresponding author/s.

## Ethics Statement

The studies involving human participants were reviewed and approved by Ethics Committee of the University of Queensland. The patients/participants provided their written informed consent to participate in this study.

## Author Contributions

YR contributed to the conception and design of the study. YR, SX, and ZT drafted the manuscript and performed the research. LS and SX contributed to analyzing the data. XH contributed to supervision, writing-review, and editing. All authors contributed to the article and approved the submitted version.

## Conflict of Interest

The authors declare that the research was conducted in the absence of any commercial or financial relationships that could be construed as a potential conflict of interest.

## Publisher’s Note

All claims expressed in this article are solely those of the authors and do not necessarily represent those of their affiliated organizations, or those of the publisher, the editors and the reviewers. Any product that may be evaluated in this article, or claim that may be made by its manufacturer, is not guaranteed or endorsed by the publisher.

## Footnotes

1
http://thoth.inrialpes.fr/people/mairal/spams/

2
https://github.com/nitishsrivastava/deepnet
