# Supplementary material for: Hierarchical Spatio-Temporal Modeling of Naturalistic Functional Magnetic Resonance Imaging Signals via Two-Stage Deep Belief Network With Neural Architecture Search
Source: Front Neurosci. 2021 Dec 8;15:794955. doi: 10.3389/fnins.2021.794955 (PMC8692564; doi:10.3389/fnins.2021.794955)
Supplement: Supplementary file 1 [file Data_Sheet_1.docx]

Supplementary Material

# Supplementary Figures


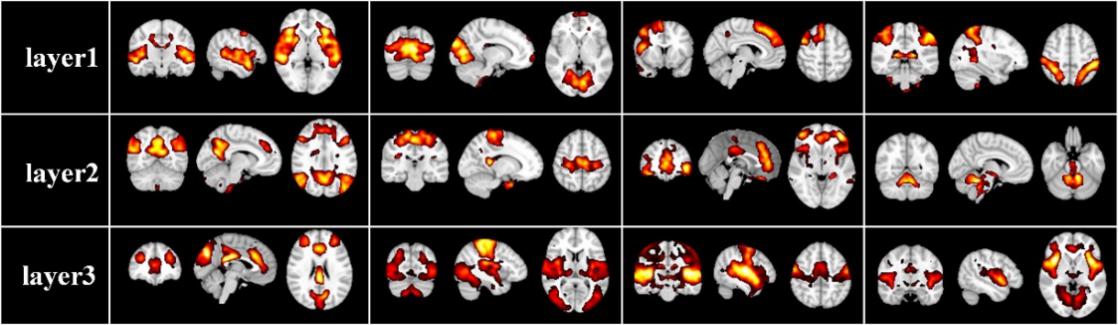


**SFigure. 1** Representative group-level functional brain networks from three layers identified by first-stage DBN model based on session B.


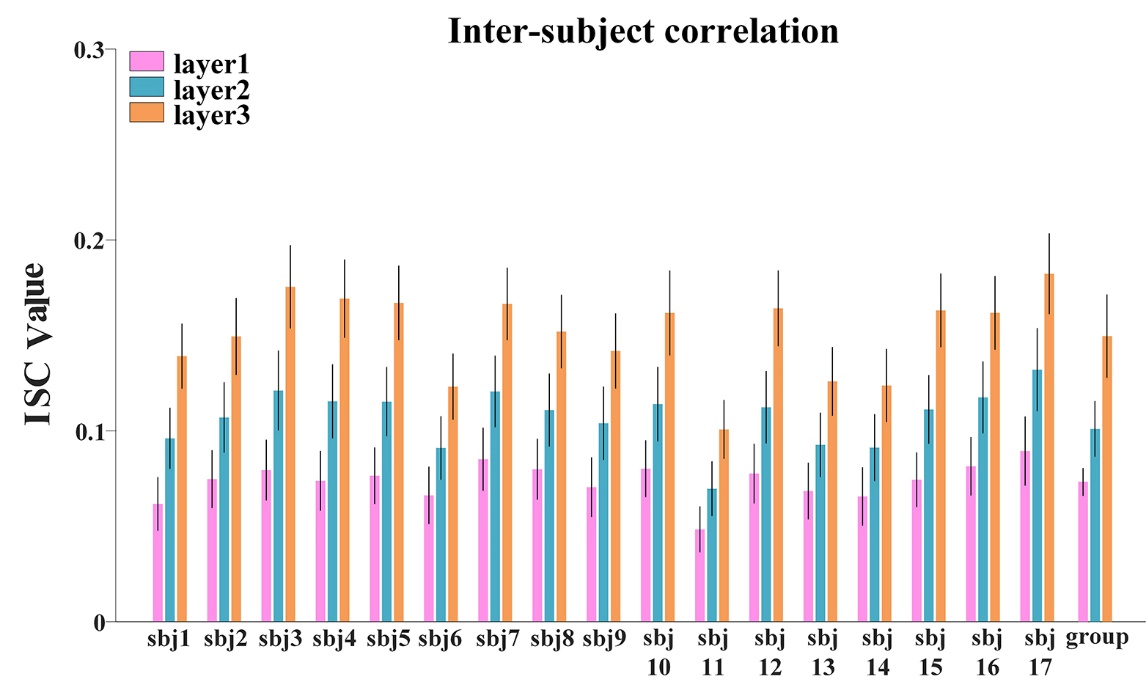


**SFigure. 2** The ISC metric in each layer at individual-level and group-level for session B. Error bar indicates standard error of mean (s.e.m).


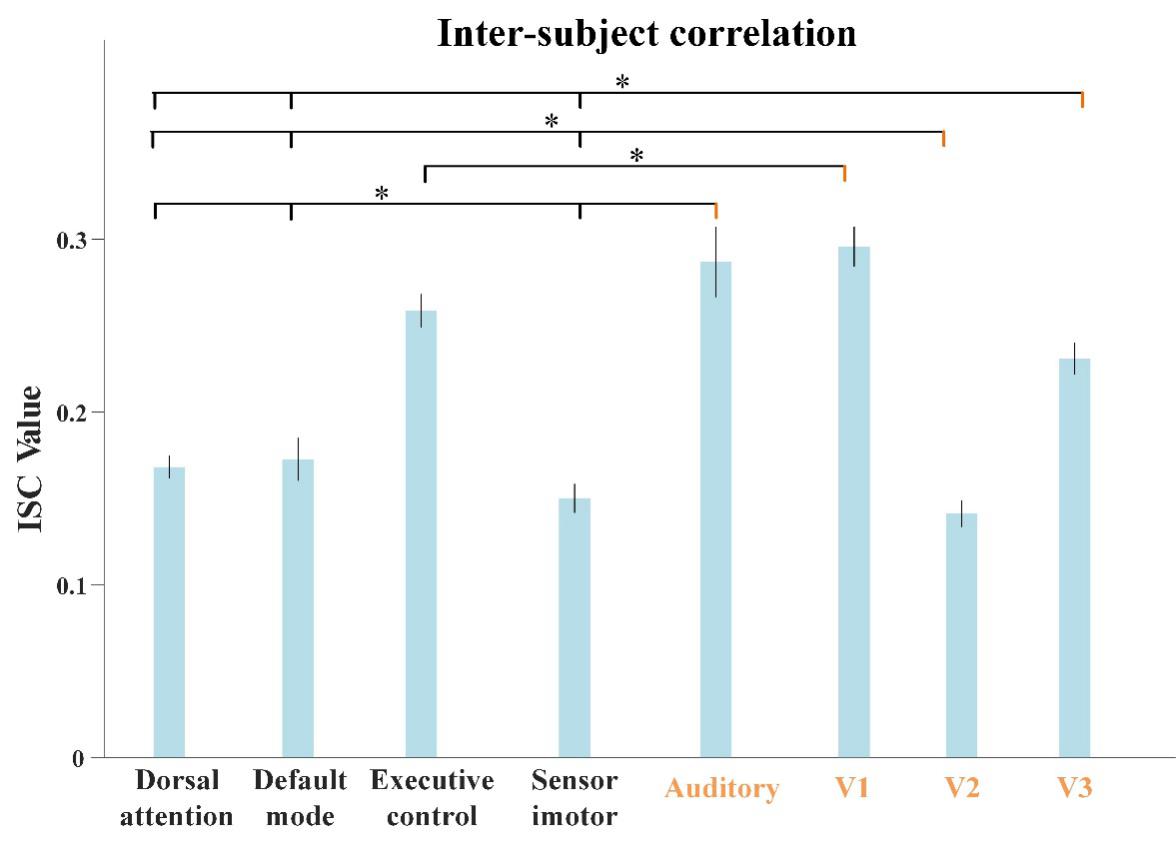


**SFigure. 3** The average ISC values from individual temporal features corresponding to each FBN for session B, including dorsal attention network, default-mode network, executive control network, sensorimotor network, auditory network, medial-visual network (V1), occipital pole-visual network (V2) and lateral visual network (V3). The error bar refers to standard error of mean (s.e.m). The statistical test was conducted by ANOVA, where * represents FDR-corrected *p*<0.001.


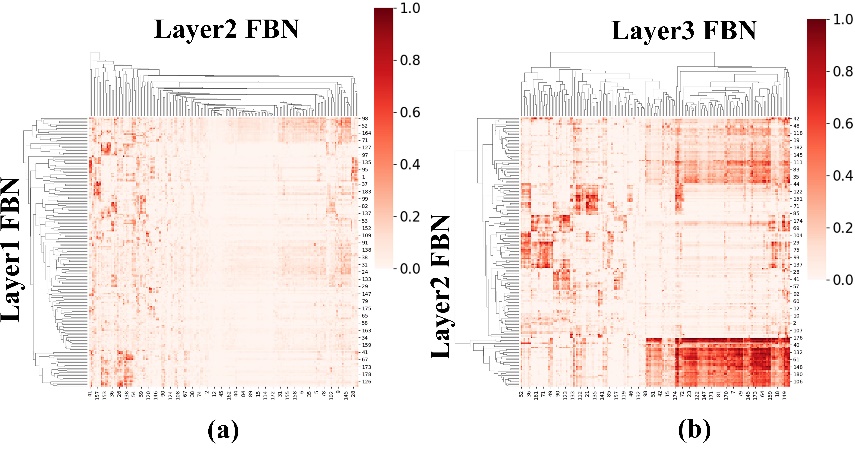


**SFigure. 4** The group-level ISR maps of spatial maps across layers for session B. (a) is the ISR between layer 1 and layer 2. (b) is the ISR between layer 2 and layer 3.


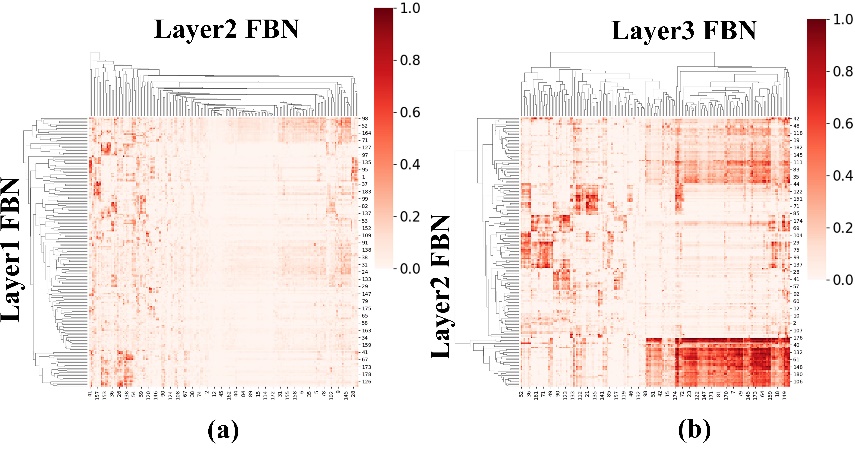


**SFigure. 5** The group-level ISR maps of temporal responses across hidden layers for session B. (A) The ISR map between layer 2 and layer 1, and (B) the ISR map between layer 3 and layer 2.


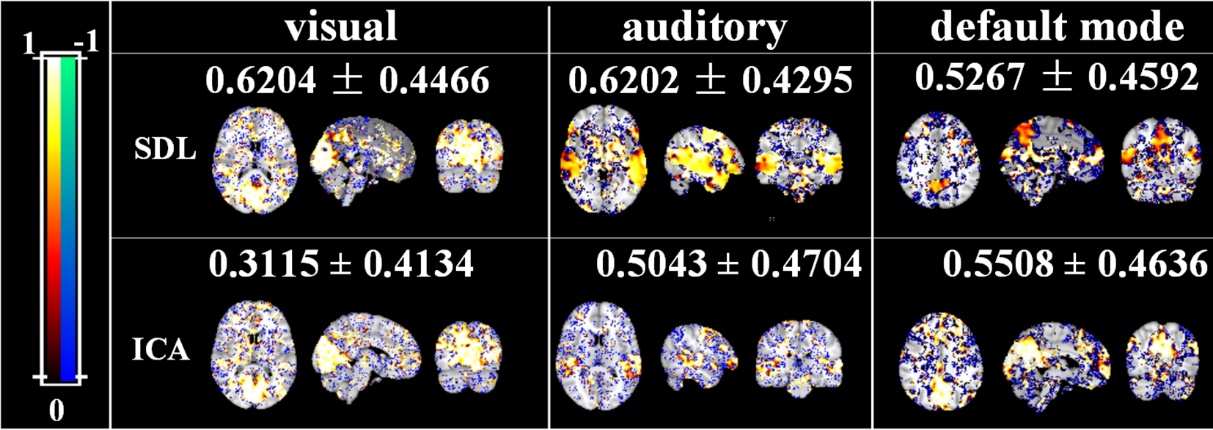


**SFigure. 6.** Brain maps of the voxel-wise ICCs of manually matching brain network using SDL and ICA methods.
